# What UK pre-hospital providers use intranasal analgesia? A survey of its current and planned use by air ambulance services in the United Kingdom

**DOI:** 10.1186/1757-7241-22-S1-P15

**Published:** 2014-07-07

**Authors:** Craig Pascoe, Scott James, Christopher Smith, Matthew Warner, Anne Weaver, David Lockey

**Affiliations:** 1 London’s Air Ambulance, The Royal London Hospital, London, United Kingdom

## Background

Intranasal analgesia (INA) has been used for many years as an alternative method of delivering pain relief in-hospital, especially in paediatric populations [1,2]. London’s Air Ambulance (LAA) is considering introducing and INA standard operating procedure (SOP) to complement their current methods of delivering analgesia pre-hospital. Little data exists on the current use of pre-hospital INA in the United Kingdom (UK). We aimed to determine what pre-hospital providers in the United Kingdom currently use or plan to introduce INA and what agents they are using.

## Methods

Air ambulance providers in the UK were identified using the association of air ambulances coverage map. Contact methods for each of the providers were identified from their individual websites. A telephone survey of the identified 19 air ambulance providers in the UK was subsequently carried out. A clinical member of the team was asked the following questions:

1. Do you currently have an SOP for INA?

2. If so which agents do you use?

3. If not do you have plans to introduce its use in the near future?

The data was collated in a Microsoft Excel spreadsheet and basic statistical analysis carried out.

## Results

Data was provided by 16 of the 19 UK services contacted. 8 (50%) currently use INA, 5 in paediatrics and 3 in paediatrics and adults. No provider used INA solely for adults. 4 (25%) are currently planning to introduce INA. 4 (25%) of the services contacted have no plans to introduce INA. 6 providers used Diamorphine, 1 ketamine and 1 diamorphine and ketamine.

## Discussion

75% of the UK air ambulance providers who responded either currently use or plan to introduce INA in the near future. This represents a large pool of clinical experience and expertise and should be used to help guide LAA in their decision to introduce INA to their current analgesia SOPs.
